# Long term follow-up to evaluate the efficacy of miglustat treatment in Italian patients with Niemann-Pick disease type C

**DOI:** 10.1186/s13023-015-0240-y

**Published:** 2015-02-27

**Authors:** Simona Fecarotta, Alfonso Romano, Roberto Della Casa, Ennio Del Giudice, Diana Bruschini, Giuseppina Mansi, Bruno Bembi, Andrea Dardis, Agata Fiumara, Maja Di Rocco, Graziella Uziel, Anna Ardissone, Dario Roccatello, Mirella Alpa, Enrico Bertini, Adele D’Amico, Carlo Dionisi-Vici, Federica Deodato, Stefania Caviglia, Antonio Federico, Silvia Palmeri, Orazio Gabrielli, Lucia Santoro, Alessandro Filla, Cinzia Russo, Giancarlo Parenti, Generoso Andria

**Affiliations:** Department of Translational Medicine-Section of Pediatrics, Federico II University, Naples, Italy; Regional Coordinator Centre for Rare Diseases, University Hospital “Santa Maria della Misericordia”, Udine, Italy; Department of Pediatrics, Regional Referral Center for Inherited Metabolic Disease, University of Catania, Catania, Italy; Department of Pediatrics, Unit of Rare Diseases, Gaslini Institute, Genoa, Italy; Unit of Child Neurology, The Foundation “Carlo Besta” Neurological Institute (IRCCS), Milan, Italy; Center of Research on Immunopathology and Rare Diseases (CMID), San Giovanni Bosco Hospital and University of Turin, Turin, Italy; Department of Neurosciences, Unit of Neuromuscular and Neurodegenerative Diseases, Bambino Gesù Children’s Hospital, IRCCS, Rome, Italy; Department of Pediatric Medicine, Division of Metabolism, Bambino Gesù Children’s Hospital, IRCCS, Rome, Italy; Department of Neurosciences, Psychology Clinic Unit, Bambino Gesù Children’s Hospital, IRCCS, Rome, Italy; Department of Medical, Surgical and Neurological Sciences, University of Siena, Siena, Italy; Department of Clinical Sciences, Polytechnic University of Marche, Ospedali Riuniti, Ancona, Italy; Department of Neurosciences, Reproductive and Odontostomatological Sciences, Federico II University, Naples, Italy

**Keywords:** Niemann-Pick disease type C, NPC, Miglustat, NB-DNJ, Substrate reduction therapy, Treatment, Therapy

## Abstract

**Background:**

Twenty-five patients with Niemann Pick disease type C (age range: 7 months to 44 years) were enrolled in an Italian independent multicenter trial and treated with miglustat for periods from 48 to 96 months.

**Methods:**

Based on the age at onset of neurological manifestations patients’ phenotypes were classified as: adult (n = 6), juvenile (n = 9), late infantile (n = 6), early infantile (n = 2). Two patients had an exclusively visceral phenotype. We clinically evaluated patients’ neurological involvement, giving a score of severity ranging from 0 (best) to 3 (worst) for gait abnormalities, dystonia, dysmetria, dysarthria, and developmental delay/cognitive impairment, and from 0 to 4 for dysphagia. We calculated a mean composite severity score transforming the original scores proportionally to range from 0 to 1 to summarize the clinical picture of patients and monitor their clinical course.

**Results:**

We compared the results after 24 months of treatment in 23 patients showing neurological manifestations. Stabilization or improvement of all parameters was observed in the majority of patients. With the exception of developmental delay/cognitive impairment, these results persisted after 48–96 months in 41 – 55% of the patients (dystonia: 55%, dysarthria: 50%, gait abnormalities: 43%, dysmetria: 41%, respectively). After 24 months of therapy the majority of the evaluable patients (n = 20), demonstrated a stabilization or improvement in the ability to swallow four substances of different consistency (water: 65%, purée: 58%, little pasta: 60%, biscuit: 55%). These results persisted after 48–96 months in 40-50% of patients, with the exception of water swallowing. Stabilization or improvement of the composite severity score was detected in the majority (57%) of 7 patients who were treated early (within 3.5 years from onset) and rarely in patients who received treatment later.

**Conclusions:**

The results of this study suggest that miglustat treatment can improve or stabilize neurological manifestations, at least for a period of time; the severity of clinical conditions at the beginning of treatment can influence the rate of disease progression. This conclusion applies particularly to patients with juvenile or adult onset of the disease.

**Trial registration:**

EudraCT number 2006-005842-35

**Electronic supplementary material:**

The online version of this article (doi:10.1186/s13023-015-0240-y) contains supplementary material, which is available to authorized users.

## Background

Niemann-Pick disease type C (NPC) is a lysosomal lipid-storage disorder, with an autosomal recessive mode of inheritance, characterized by defective intracellular lipid trafficking, and secondary accumulation of free cholesterol, sphingosine and glycosphingolipids in the lysosome-late endosome compartment of different tissues and organs. The brain is particularly affected by this pathological accumulation.

NPC is estimated to affect 1 in 150,000 live births and can be genetically caused by mutations in either the *NPC1* gene (more than 95% of all NPC cases) or the *NPC2* gene (the remaining 5%).

NPC is clinically characterized by a wide spectrum of visceral and neurological signs and symptoms, with variable age of onset [1]. Classically, NPC disease has been classified in many phenotypes based on the patient’s age of onset, independently from whether the first signs were visceral or neurological [1]. More recently, categorization of patients has been based on the age at onset of the first neurological sign [2].

Neurological findings in NPC include vertical and horizontal supranuclear gaze palsy, ataxia, dysarthria, dysphagia, dystonia, seizures, progressive dementia, psychiatric syndromes and gelastic cataplexy. These manifestations have a continuous, unbroken progression, consistently more rapid in patients diagnosed in early childhood, compared with those with later onset [3]. Visceral symptoms include hepatosplenomegaly and pulmonary infiltrates.

No treatment was available for NPC patients until 2004, when miglustat (NB-DNJ, Zavesca®, Actelion Pharmaceuticals, Ltd) was proposed for the treatment of the disease [4]. Miglustat is a small iminosugar molecule able to cross the blood–brain barrier and to reversibly inhibit glucosylceramide synthase, which is the first enzyme in glycosphingolipid synthesis.

The efficacy of miglustat on progression of neurological manifestations has been studied in NPC patients enrolled in international clinical trials and observational studies. Data from one-year treatment of juvenile and adult NPC patients enrolled in an international standard randomized controlled trial [5] suggested that miglustat improves or stabilizes several neurological manifestations.

Data from long-term treatment of ten affected children enrolled in a parallel, uncontrolled study [6] and from patients participant to further studies [7-13] confirmed the previous results and extended them to pediatric patients, also suggesting that the magnitude of the effect was greater in juvenile and adult subjects [14].

Miglustat has been approved by the European Agency of Medicines in 2009 for the treatment of neurological manifestations in NPC patients. Nonetheless experience is still limited and long-term efficacy is unknown.

We report the results of an independent multicenter clinical trial of miglustat treatment in Italian patients, carried out from 2007 to 2009, before the drug was approved in Europe, and from long-term follow-up, lasting up to 48–96 months.

## Methods

### Patients

Twenty-five NPC patients were enrolled at 11 Italian clinical centers. Enrolled patients were diagnosed on the basis of biochemical tests (filipin staining and LDL-cholesterol esterification) and/or molecular analysis of *NPC1* or *NPC2* genes.

Patients were classified in four clinical forms based on the age at onset of the first neurological symptoms and signs [2]: Early Infantile (EI): ≤ 2 years of age; Late Infantile (LI) : > 2 and ≤5 years of age); Juvenile (J): > 5 and ≤16 years of age; Adult (A): >16 years of age.

The disease phenotype of two patients was exclusively visceral (V) as they did not show any neurological manifestation at start of treatment. One of them was diagnosed at 0.6 years of age for a prolonged cholestatic jaundice and hepatosplenomegaly (V14); the other one was the younger brother (V23) of a patient already showing the LI form and was diagnosed at 1.3 years, due to the presence of splenomegaly.

One patient (J15) (who was the sister of a juvenile patient) was treated very early, at the first appearance of neurological manifestations, consisting of minimal slurred speech. Her phenotype was classified as a J form.

Table 1 summarizes patients’ cohort characteristics. We enrolled familial cases in 4 groups of siblings (J15 and J16; LI08 and V23; A02 and A19; J06, J12 and J13). Additional file 1 summarizes individual patients’ phenotype, duration of miglustat treatment and follow-up, and latency between onset of neurological manifestations and start of treatment. Some patients have been reported in the literature in the meantime (J15, J16, SI22, LI09 in Fecarotta et al. [15]; LI08, V14, V23, in Di Rocco et al. [9]; LI 21, J11, LI05, J07, J12, J13 and A01 in Ginocchio et al. [13]).

**Table 1:** **Patients’ demographic, molecular and clinical features**

	**Early-infantile phenotype**	**Late-infantile phenotype**	**Juvenile phenotype**	**Adult phenotype**	**Visceral phenotype**	**All patients**
***Number of patients***	2	6	9	6	2	25
***Gender***						
male	2	1	3	2	1	9
female	0	5	6	4	1	16
***Number of Adult patients (Age >16 years atstart of treatment)***	0	1	2	6	0	9
***Number of Pediatric patients (Age*** **≤** ***16 years at start of treatment)***	2	5	7	0	2	16
***Age at diagnosis (years)***						
Mean (SD)	1.63 (1.37)	6.69 (4.22)	11.38 (2.55)	28.42 (8.55)	0.94 (0.51)	12.73 (10.69)
Range	0.66 - 2.60	0.25 - 10.6	7.70 - 16.00	18.00 - 43.83	0.58 - 1.30	0.25 - 43.83
***Age at enrollment (years)***						
Mean (SD)	3.0 (0.06)	10 (4.18)	16 (3.12)	32 (8.89)	2.0 (0.53)	16 (11.72)
Range	2.66 - 2.75	3.10 - 15.66	10.3 - 19.60	18.83 - 43.83	2.33 - 1.58	1.58 - 43.83
***Age at start of treatment (years)***						
Mean (SD)	1.83 (1.3)	9.53 (4.45)	15.31 (3.5)	31.93 (8.95)	1.09 (0.72)	15.69 (11.59)
Range	0.91 - 2.75	3.0 - 16.7	9.41 - 19.60	19.00 - 43.83	0.58 -1.60	0.58 - 43.83
***Time between onset of first neurological manifestation and start of treatment (LAG) (years)***						
Mean (SD)	0.71 (0.66)	6.22 (4.23)	6.24 (4.26)	8.74 (3.84)	0	5.97 (4.49)
Range	0.66 - 0.75	1.00 - 12.70	0.41 - 11.83	3.00 - 13.80	0	(0–13.80)
***Number of patients on miglustat treatment at enrollment***	1	2	5	2	1	11
***Duration of previous miglustat treatment at enrollment (months)***						
Mean (SD)	11.0 (14.85)	4.0 (7.27)	11.0 (13.0)	9.0 (12.60)	11.0 (15.56)	9.0 (11.13)
Range	0 - 21	0 - 18.0	0 - 31.0	0 - 23.0	0 - 22.0	6.0 - 31.0
***Duration of miglustat treatment (months)***						
Mean (SD)	72.0 (33.94)	67.0 (12.5)	76.0 (16.7)	68.0 (22.34)	72.0 (16.97)	71.0 (22.11)
Range	48.0 - 96.0	48.0 - 84.0	54.0 - 96.0	48.0 - 96.0	60.0 - 84.0	48.0 - 96.0
***Molecular analysis***						
NPC1 gene mutations (n. of patients)	1	6	9	5	2	23
NPC2 gene mutations (n. of patients)	1	0	0	1	0	2

### Study design

Enrolled patients represented all known NPC Italian patients in the years 2007–2009. A subset of 11 patients was already on miglustat treatment at enrollment (the drug was paid from the National Health service as an off-label prescription). As a consequence, this study was partly conceived as a single arm, open label, clinical trial and partly as an observational study, to evaluate the efficacy of miglustat therapy in NPC patients. A long-term follow-up, up to 48–96 months of treatment was established in enrolled patients.

The study was carried out in accordance with criteria and procedures outlined in the declaration of Helsinki and in compliance with ICH Good Clinical Practice guidelines. Local Ethics Committee approval and written informed consent from all patients or their legal representatives were obtained before enrollment.

The study was supported by the Italian Medicines Agency (Agenzia Italiana del Farmaco, AIFA, Rome, trial number FARM59T23W; EUDRACT number 2006-005842-35) and the trial started in 2007, before miglustat was commercially approved for the treatment of neurological manifestations in NPC disease.

### Treatment regimen

Patients over 16 years of age received orally 200 mg t.i.d. of miglustat (NB-DNJ, Zavesca®, Actelion Pharmaceuticals, Ltd), if tolerated, with dosage reduction in the case of drug-related adverse events, while younger patients received the drug with dosage adjusted to body surface area, corresponding to a range of 220–300 mg/m^2^/day (total daily dose between 100 and 600 mg/day). One pediatric patient (LI17) who had already started treatment before enrollment in the study, (age at start of treatment 10.9 years), continued to receive a dosage similar to adult patients (600 mg/day), as administered before enrollment. Three patients over 16 years (LI25, J13 and J20) who did not tolerate the regimen for adults of 600 mg/day, received a reduced dosage, between 300 and 500 mg/day corresponding to a dosage per surface area similar to that of pediatric patients.

### Methods

Primary endpoints were chosen to clinically evaluate the efficacy of miglustat on neurological signs and symptoms and were defined as stabilization or improvement of neurological involvement including swallowing abnormalities. Safety was monitored by registration of adverse events and changes in physical or biochemical parameters. The evaluated parameters were gait abnormalities, dysmetria, dystonia, dysarthria, developmental delay/cognitive impairment and dysphagia. The outcome was established comparing the assessment at baseline with the last available evaluation in each patient.

In 14 patients who had not been treated with miglustat at the enrollment, the baseline was defined as the neurological assessment at the start of treatment and data were prospectively collected; in 11 patients who were already on treatment at the enrollment in the trial, the baseline data were retrospectively retrieved.

### Rating scale of neurological parameters

Each parameter was evaluated on a 4- or 5-point scale and the overall severity of the neurological status, including dysphagia, was summarized using a mean composite severity score (MCSS). The original scoring was then modified by assigning a score from 0 (best) to 1 (worst), to give equal weight to each parameter (Additional file 2).

Gait abnormalities, dysmetria, dystonia, dysarthria and developmental delay/cognitive impairment were assessed by the same neurologist at each participating center, assigning a 4-points score.

Additionally, developmental delay/cognitive impairment was also assessed in selected patients using formal psychometric tests as Griffith’s mental developmental scale and Wechsler-Bellevue scale (WPPSI, WISC-R, WAIS-R). Raven’s Progressive Matrices Test was used in a single patient (J11) with severe dysarhtria to measure non-verbal reasoning. Psychometric tests were chosen according to patient’s age and clinical conditions. In each patient the same test was administered by the same specialist, if appropriate for age and clinical conditions, to compare subsequent assessments.

When psychometric evaluations were available, patients were categorized according to the classification of the American Association of Mental Retardation, the Diagnostic and Statistical Manual of Mental Disorders, Fifth Edition (DSM-5) (Additional file 2).

Dysphagia was clinically assessed, assigning a score to patient’s ability of swallowing four substances of different consistencies (water, purée, little pasta and biscuit), according to a 5-point score, in agreement with methods used in previous international clinical trials [5]. Dysphagia was also assessed using videofluoroscopic swallowing study (VFSS) in 4 pediatric patients, to provide additional information on patterns of impairment of the swallowing mechanism and to detect aspiration [15].

### Data analysis

The two patients with the visceral phenotype were evaluated separately from the other 23 neurologically symptomatic patients.

Absolute values and changes from baseline in individual scores were analyzed using descriptive statistics.

For gait abnormalities, dysmetria, dystonia and dysarthria, we did not evaluate any change from baseline for those patients who had the most severe score at baseline (completely invalidating the specific function) and who did not show any improvement following miglustat treatment. Neither gait abnormalities nor dysarthria were evaluated in a patient (EI22) younger than 12 months at baseline because physiologically unable to walk and speak.

For developmental delay/cognitive impairment, when finer psychometric evaluations were available, two patients (LI09 and LI17) within the severe/invalidating category were judged evaluable for changes from baseline, even if they remained in the same most severe category after treatment, because improvement, stabilization or deterioration were actually based on the two subcategories of severe and profound intellectual disability, respectively. Similarly, patients within the first category for developmental delay/cognitive status (absence of cognitive/psychomotor impairment) were defined as stable, improved or deteriorated based on the two subcategories of absent and borderline intellectual disability, respectively.

A mean composite severity score (MCSS) was also calculated, including all parameters, as the mean of the individual modified scores in each patient, at baseline and at each follow-up visit. In patient EI22 the MCSS was calculated at baseline assigning the modified score to dystonia, dysmetria, developmental delay/cognitive impairment and dysphagia and the mean of modified scores was calculated on the 4 evaluated parameters, while all the subsequent evaluations were performed on all six parameters.

Considering that the most severe swallowing difficulties had been detected for liquids in all patients, we scored the dysphagia parameter in the MCSS referring to the swallowing of water.

## Results

### Severity scores: distribution at baseline and change after treatment

Severity score for each parameter was assessed at baseline in 23 patients with neurological manifestations. Severity score distribution showed that clinical conditions of patients at baseline were largely heterogeneous (Additional file 3 A).

We studied the effects of miglustat, assessing the change of severity for each parameter. Results were summarized as the proportion of patients showing improvement (I), stabilization (S) or deterioration (D) for each parameter from baseline to the last available evaluation, as previously defined.

Among evaluable patients, most of them showed improvement or stabilization of the severity score after 24 months of treatment for gait abnormalities (I = 7%; S = 79%), dystonia (I = 10%; S = 70%), dysmetria (I = 11%; S = 50%), dysarthria (I = 13%; S = 56%), developmental delay/cognitive impairment (I = 10.5%; S = 58%) and dysphagia (water dysphagia: I = 20%; S = 45%). With the exception of developmental delay/cognitive impairment, these results persisted in 41 – 55% of the patients (in 55% for dystonia, 50% for dysarthria, 43% for gait abnormalities and 41% for dysmetria) after 48–96 months of treatment (Additional file 3 B).

While stabilization was more prevalent, improvement of some neurological parameters was seen in few patients. We did not consider as improved one patient (EI22) that was not evaluated at baseline for gait abnormalities and dysarthria (as he was younger than 12 months) and that acquired ambulation and speech during the miglustat treatment. In this patient, who showed the severe p.E20X homozygous mutation in the *NPC2* gene and had a severe neurological involvement at baseline, psychometric tests surprisingly evidenced a sustained improvement, with progress in reaching psychomotor milestones. While a progressive decline over time was expected, according to the natural history of the disease, the Developmental Quotient as assessed by Griffith's mental developmental scale changed from 45 (chronological age, CA = 11 months) to 63 after 24 months of therapy, (CA = 38 months) and the Intelligence Quotient as assessed by WPSSI was 61 after 36 months of treatment (CA = 4 years). However, deterioration of the mental status was observed in the long-term follow-up and IQ was measured as 52 after 48 months (at CA = 5 years).

Among enrolled patients, three had a severe dysphagia at enrollment and food and drug were administered using a percutaneous enterogastrostomy (PEG) (Additional file 3 C). Using qualitative tests for the clinical assessment of swallowing in 20 evaluable patients, we showed improvement or stabilization of ability to swallow 4 different substances in most of patients (water: I + S = 65%, purée: I + S = 58%, little pasta: I + S = 60%, biscuit: I + S = 55%) after 24 months of therapy; these results persisted in 40 – 50 % of patients after 48–96 months, with the exception of water swallowing (Additional file 3D).

We observed a late severe worsening of dysphagia, requiring the application of a PEG, in a single late-treated patient (A02), after years of stabilization during miglustat treatment.

Video-fluoroscopic swallowing study, additionally used in 4 children treated with miglustat for 3 years or more, showed a clear-cut improvement in 3 patients with abnormalities at baseline. The patient (J15) who had not shown any swallowing abnormality at baseline remained stable [15] after 84 months of treatment.

### Clinical course evaluated by the mean composite severity score (MCSS)

We calculated a MCSS in every patient at each visit during the long-term follow-up to summarize the course and evolution of neurological manifestations. Patients were stratified based on three parameters: phenotypic form, latency between neurological onset and start of treatment (LAG) and severity of clinical conditions at treatment baseline, to evaluate which parameters could affect the response to treatment.

**Figure 1:**
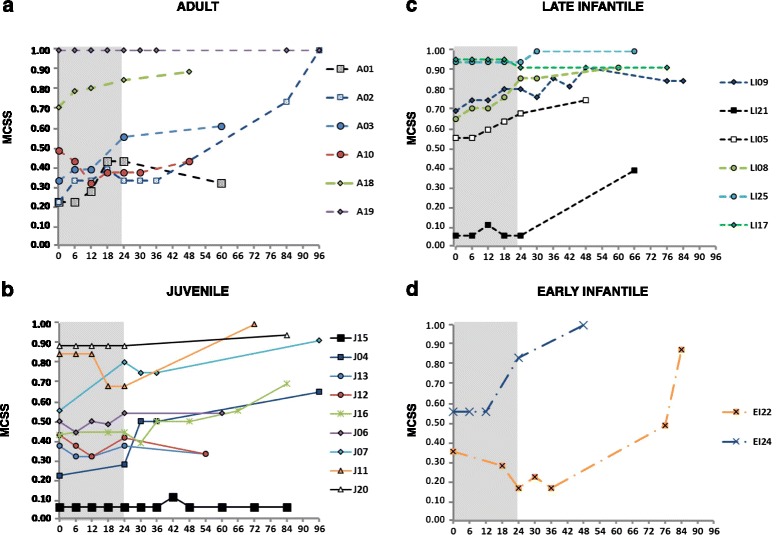
**Evolution over time of the mean composite severity score (MCSS) of neurologically symptomatic patients during miglustat treatment.** Patients with **a)** adult (n = 6), **b)** juvenile (n = 10), **c)** late infantile (n = 5) and **d)** early infantile (n = 2) phenotypes.

Long-term stabilization or improvement of the MCSS was shown in some patients with adult (1/6) and juvenile (3/9) phenotypes (Figure 1). The two patients with visceral phenotypes (V14 and V23) who were treated with the aim to prevent the onset of neurological manifestations, did not show any neurological symptom and sign after 60 and 84 months of treatment, respectively (data not shown) [9].

Patients with early onset of the disease showed neither long-term stabilization nor improvement, even if they responded well to treatment in the first 24 months (Figure 1).

**Figure 2:**
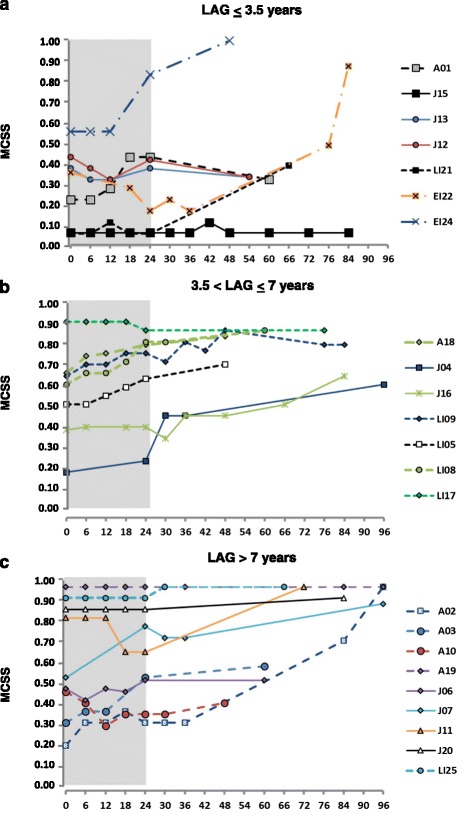
**Evolution over time of the mean composite severity score (MCSS) during miglustat treatment in different groups of patients, classified for the latency between the onset of neurological manifestations and start of therapy (LAG).** Patients with **a)** LAG ≤ 3.5 years (n = 7); **b)** LAG between >3.5 and ≤ 7 years (n = 7); **c)** LAG > 7 years (n = 9).

Improvement or stabilization of the MCSS were noticed in the majority (5/9; 56%) of patients who received an early treatment (less than 3.5 years from the onset), but just in a single patient (A10) who received a late treatment (1/16; 6.25%) (Figure 2).

**Figure 3:**
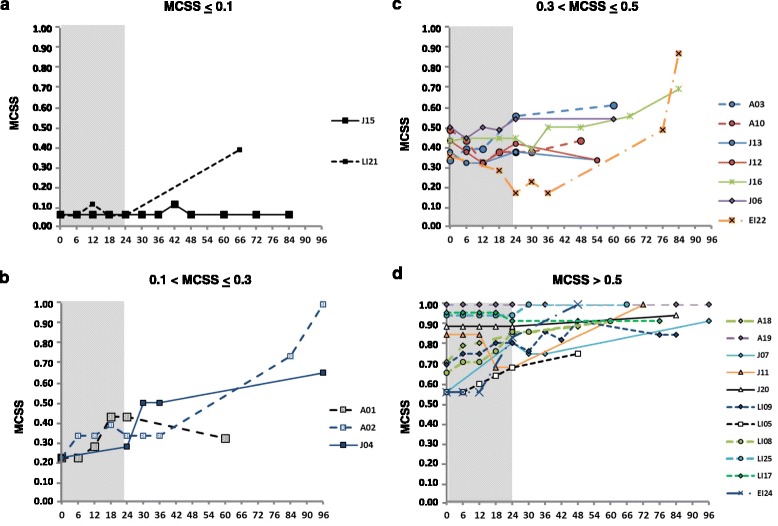
**Evolution over time of the mean composite severity score (MCSS) during miglustat treatment in different groups of neurologically symptomatic patients, classified for the MCSS at baseline.** Patients with **a)** MCSS ≤ 0.1 (n = 2), **b)** patients with MCSS between > 0.1 and ≤ 0.3 (n = 3), **c)** patients with MCSS > 0.3 and ≤ 0.5 (n = 7) and **d)** patients with MCSS > 0.5 (n = 11).

The severity of clinical conditions at start of treatment was related to the progression rate. All patients with the most severe neurological involvement (MCSS > 0.5) did not show any improvement, while patients who were treated even with very mild neurological involvement were stable after 7 years of treatment. Progression of the disease was variable in patients with low and intermediate MCSS (Figure 3).

We particularly analyzed the juvenile phenotype group, which was the most numerous one, and noted that, even among patients with a similar severity score at baseline (between 0.1 and 0.50), those who received an early treatment (<3.5 years from the onset of neurological signs) showed a better response than those who were treated later (Additional file 4).

### Comparison of miglustat efficacy in siblings

We analyzed two couples of siblings who presented a discrepancy in the LAG category and in the severity of conditions at baseline. In the first pair (J15 and J16), one patient started the treatment immediately at the onset of very mild neurological signs and symptoms; she remained stable and did not develop further neurological manifestations to the end of follow-up. At her same age, her older sister, despite miglustat treatment for 5 years, showed a serious neurological involvement, with a MCSS of 0.56 (Additional file 5 A). Similarly, in the other pair of sibs (LI08 and V23), the latter, who received the diagnosis before the onset of neurological symptoms, based on his sister’s infantile phenotype and the presence of isolated splenomegaly, started the treatment at 19 months of life and did not show any neurological involvement until the end of the trial [9]. The older sister (LI08) started the treatment with a LAG of 5.25 years and her clinical conditions progressively worsened (Additional file 5 B).

We studied another couple of sibs who were in the same LAG category > 7 years (A02 and A19). Both patients showed a similar progression of the disease, even though one of them had a milder phenotype at the start of treatment (Additional file 5 C).

Finally, two (J12 and J13) of three sisters were both treated early (within 3.5 years from onset), and showed a similar stabilization of their conditions. Differently, the third sibling (J06) who started treatment with a longer latency (LAG > 7 years) showed a slow progression of the disease (Additional file 5 D).

### Safety evaluation

During the treatment, the following adverse events were detected in different patients: epistaxis and thrombocytopenia, insomnia, leukopenia, behavioral problems, extrapyramidal symptoms, tremors, hypertransaminasemia, weight loss, diarrhea.

Weight loss and diarrhea were the most common adverse events during miglustat treatment.

One serious adverse event was recorded in a 16 years old girl affected by epilepsy, who was hospitalized for uncontrolled seizures. Appropriate anticonvulsant therapy was prescribed. The patient was discharged after the control of seizures was reached. According to the investigator, this event was related to the disease and not to the study treatment.

None of the reported adverse events was life threatening.

## Discussion

We describe an Italian cohort of 25 NPC patients who were treated with miglustat for 4–8 years.

We collected clinical data for the first 24 months of therapy, partly prospectively in patients enrolled in an independent clinical trial and partly retrospectively in patients already on miglustat treatment; a prospective observational study was continued up to 24–72 months of further follow-up. The clinical trial started in parallel with the first sponsored clinical trials for the commercial approval of miglustat [5,6].

### Features of patients’ series

We report data from 11 patients with neurological manifestations younger than 16 years of age at start of miglustat therapy, who were treated for 48–96 months. To our knowledge such a long term follow-up has not been previously reported in a large pediatric case series.

The clinical experience of long-term miglustat treatment in children before 16 years of age is still limited in previous studies [7-13] since some of these described single cases or small series of children with heterogeneous conditions, treated for variable periods [7,9,11,13].

Large cohorts of NPC children were previously reported [6,8,10,12], but the follow-up was shorter than 36 months, except in three patients from the series reported by Pineda [8] and in one patient reported by Hèron [10].

Two patients had only visceral signs and started treatment before miglustat had been approved only for the treatment of neurological manifestations.

### Set up of a severity scoring scale

In NPC disease there are no biochemical markers, used to evaluate the efficacy of treatment, that clearly correlates with the severity of clinical manifestations. We set up a clinical scoring scale for a series of neurological parameters such as gait abnormalities, dysmetria, dystonia, dysarthria developmental delay/cognitive impairment and dysphagia. This severity score system was designed before the publication of various studies using the NPC disability score or its modification [16]. Our original score is slightly different, but actually equivalent to the NPC disability score, since it evaluated similar neurological areas and allowed the assessment of disease evolution over time.

### Evolution of neurological manifestations in the case series

The 23 neurologically symptomatic patients showed an extreme heterogeneity in terms of clinical conditions at start of treatment, even in the same phenotypic subgroup. After miglustat treatment, in the short-term follow-up (24 months) we detected the stabilization or improvement of all parameters in the majority of patients (Additional file 3). After such a short-term observation, these results were consistent with those previously reported in the literature [5,6,8].

At the end of the long-term follow-up (48–96 months after start of treatment), improvement or stabilization was still present in approximately half of patients, with the exception of developmental delay/cognitive impairment (Additional file 3). The results were striking encouraging for dysphagia, as previously reported [15]. The improvement or stabilization of swallowing ability for a long time might represent a major clinical benefit for NPC patients, in terms of immediate impact on the quality of life and decreased risk of aspiration, with a consequent longer survival [17].

### Parameters affecting the response to miglustat treatment

In addition to the evaluation of each parameter in the overall population of neurologically symptomatic patients, we tried to examine the evolution of the CNS involvement in each patient, that is more relevant to understand the efficacy of miglustat treatment. Similarly to previously reported studies, we calculated a MCSS, equivalent to the NPC composite disability score [16], to summarize the clinical evolution of each patient during treatment.

Given the wide variability of the clinical course in the patients’ population, our study was aimed at understanding which parameters could affect the response to treatment. We stratified the patients’ population based on clinical phenotype, MCSS at baseline and latency between onset of neurological manifestations and start of treatment (LAG).

Consistently with previously reported data [14], patients with late-onset phenotypes, such as Juvenile or Adult ones, showed better responses to treatment than patients with an early onset phenotype, who worsened in the long-term follow-up, even when they had shown improvement or stabilization of neurological manifestations in the first 24 months of therapy. This initial improvement or stabilization was associated with a shorter interval between the onset of symptoms and the start of treatment in three patients (LI21, EI24, EI22), who already presented obvious neurological manifestations, as discussed later.

As expected, patients who showed less severe symptoms when they started miglustat treatment responded better than patients who already showed more severe clinical conditions. Moreover, the patients treated earlier (within 3.5 years from the onset) had better response to treatment than those who had longer time intervals between the onset of neurological manifestations and the start of treatment. This was particularly evident by analyzing a larger phenotype subgroup, as that of juvenile patients (Additional file 4), also including pairs of sibs. The disease progression was stopped for more than 7 years in one of two sibs (J15), who was treated at the first appearance of minimally slurred speech.

Even among five juvenile patients who had similar conditions at start of treatment, with a MCSS between 0.1 and 0.5, two early treated sisters improved (J12, J13), while three patients treated later (J16, J06, J04) (including the third sister of two early treated sibs), showed progressive deterioration.

These data suggested that, even with comparable clinical conditions at the start of treatment, the latency between the onset of symptoms and the beginning of the treatment was the most important parameter to prevent a rapid deterioration and lead to a longer stabilization of neurological manifestations.

### Treatment of asymptomatic patients

At the moment, there is still an open question on the decision of starting treatment with miglustat in neurologically symptom-free patients and if it may delay the onset and progression of neurological manifestations. When we enrolled in our trial two NPC patients showing visceral phenotype or sibs without neurological manifestations, miglustat had not been approved for NPC treatment, and neither clinical indication nor efficacy on visceral symptoms were known.

In our case series miglustat treatment was started in a child showing isolated splenomegaly, treated even before the onset of any neurological manifestation (V23) [9] because he was the brother of a symptomatic child.

Current recommendations on miglustat treatment state that a confirmed NPC diagnosis should not be taken as the only indication for immediate miglustat therapy, since neurological, psychiatric and/or cognitive manifestations can take a long time to appear [18]. However, it is difficult to decide which is the appropriate time to start treating a still symptom-free sib of an already symptomatic patient. Even considering an intrafamilial phenotypic variability, the absence of neurological manifestations in V23 at 6.5 years of age, suggested the efficacy of miglustat to prevent neurological manifestations for a long time in neurologically asymptomatic patients. Therefore, we think that the treatment of sibs should be considered on a case-by-case basis, taking into account parents’ expectations and pressures to start miglustat treatment as soon as possible.

A still open issue remains the treatment of infants presenting with isolated visceral manifestations without a family history, as our case V14. The aim of miglustat treatment in these patients should be the prevention of the onset of neurological symptoms and signs, but we think that monitoring the response to treatment is currently impossible in absence of a good biomarker correlating with neurological involvement.

### Efficacy of treatment in the NPC2 subset of patients

Until now information is scarce on the efficacy of miglustat treatment in patients with proven *NPC2* mutations, because, to the best of our knowledge, it is limited to single experiences or anecdotal reports [10].

We treated from 11 months of age a 9-year-old child (EI22) with a homozygous nonsense mutation (p.E20X) of the *NPC2* gene, after a short latency of 5 months from the onset of first neurological signs. After miglustat treatment he showed a progressive improvement of the developmental/intelligence quotient, which persisted after 36 months of treatment. Then, his neurological status progressively deteriorated in the long-run, but improvement of swallowing ability was sustained over time. The improvement of dysphagia was associated to improvement of growth parameters [15] (and perhaps to a longer survival, compared to his older affected brother, who died at 10 months of age).

The observed evolution in this patient is quite different from the natural history in infantile patients with the p.E20X mutation of *NPC2* gene, who usually show a serious, precocious and rapidly progressive neurological involvement [19,20]. Whether early initiation of treatment in this patient influenced the clinical course has to be confirmed by further cases.

We also treated an adult patient (A03), showing the c.26 T > C (p.L9P) homozygose mutation of the *NPC2* gene. Patients with missense mutations of the *NPC2* gene showed variable phenotypes, including juvenile and adult onset forms [19,20]. Miglustat treatment was started very late, after a latency of more than 10 years from the neurological onset. The clinical course was characterized by a progressive neurological deterioration, similarly to adult patients with mutations in *NPC1*, who were treated late. Our data in patients with mutations in *NPC2*, although limited to single cases, suggested that the outcome of patients with *NPC2* gene mutations could not differ from that of the *NPC1* group. Similarly to them the response to miglustat therapy could be affected by the same parameters, such as phenotype, severity of conditions at start of therapy and latency between the onset of neurological manifestations and the start of treatment.

### Limitations and biases

We are aware of limitations and biases of the present study, due to its design and to the clinical heterogeneity of patients.

First, this study was partly interventional, and partly observational, as some patients had already started treatment with the investigated medicinal product at the moment of the official enrollment in the trial.

Enrolled patients were heterogeneous, both genetically (23 patients had mutations of *NPC1* and 2 patient had mutations of *NPC2*), and phenotypically (we included patients with variable age of presentation). Age range was also wide and patients included in the same phenotypic category had heterogeneous clinical conditions at enrollment, from pre-symptomatic to very severe conditions.

Our study also lacked a control group and the evolution of severity scores before treatment was unknown.

## Conclusions

Our data suggested that an early diagnosis and treatment were important to benefit from miglustat therapy, particularly in juvenile and adult onset disease. Consistently with the ability of miglustat to cross the blood–brain-barrier, our data supported the published evidence that the substrate reduction has some efficacy in modifying the natural course of neurological manifestations in NPC disease [5-15].

Finally, considering that natural history of NPC disease is characterized by a linear progression of neurological symptoms [3,21], our results indicated that later onset phenotypes, milder neurological manifestations and earlier start of miglustat treatment were associated with stabilization or even improvement of clinical conditions, at least for some time.

## Additional files

Additional file 1:
**Patients’ phenotype and genotype, duration of miglustat treatment and follow-up, latency between onset of neurological manifestations and start of treatment.**
Additional file 2:
**Severity rating scale of neurological manifestations and dysphagia.**
Additional file 3:
**Distribution of severity score of five neurological parameters at baseline and after 24 and 48–96 months of treatment.** **A:** The severity score distribution for gait abnormalities, dystonia, dysmetria, dysarthria and developmental delay/cognitive impairment showed heterogeneous clinical conditions in enrolled patients at baseline **B:** Modification of severity score of five neurological parameters, compared to baseline, after 24 and 48–96 months of treatment. After 24 months of treatment most patients showed improvement or stabilization of the severity score for gait abnormalities, dystonia, dysmetria, dysarthria and developmental delay/cognitive impairment. **C:** Distribution of severity score for swallowing of four different substances at baseline and after 24 and 48–96 months of treatment. The severity score distribution for swallowing ability showed heterogeneous clinical conditions in enrolled patients at baseline. **D:** Modification of severity score for swallowing of four different substances, compared to baseline, after 24 and 48–96 months of treatment. After 24 months of treatment most patients showed improvement or stabilization of the ability to swallow four substances with different consistencies. Abbreviations: I = improvement; S = stabilization; D = deterioration. Additional file 4:
**Evolution over time of the mean composite severity score (MCSS) during miglustat treatment in juvenile patients, based on the latency between the onset of neurological manifestations and start of therapy (LAG).** Patients with **a)** LAG ≤ 3.5 years (n = 3); **b)** LAG >3.5 years (n = 6). Additional file 5:
**Evolution over time of the mean composite severity scores (MCSS) in groups of siblings.** In sections **a)** and **b)** siblings were treated with different latency between the onset of neurological manifestations and start of therapy (LAG); in section **c)** siblings were both treated with LAG > 7 years. In section **d)** both patients J12 and J13 were treated early (LAG < 3,5 years), while patient J06 had a longer latency.

## Abbreviations

NPC: Niemann-Pick disease type C
EI: Early infantile
LI: Late infantile
J: Juvenile
A: Adult
V: Visceral
MCSS: Mean composite severity score
CA: Chronological age
VFSS: Videofluoroscopic swallowing study
I: Improvement
S: Stabilization
D: Deterioration
PEG: Percutaneous enterogastrostomy
LAG: Latency between neurological onset and start of treatment
