# Supplementary material for: Long term follow-up to evaluate the efficacy of miglustat treatment in Italian patients with Niemann-Pick disease type C
Source: Orphanet J Rare Dis. 2015 Feb 27;10:22. doi: 10.1186/s13023-015-0240-y (PMC4359492; doi:10.1186/s13023-015-0240-y)
Supplement: Additional file 1: — Patients’ phenotype and genotype, duration of miglustat treatment and follow-up, latency between onset of neurological manifestations and start of treatment. [file 13023_2015_240_MOESM1_ESM.pdf]

Additional file 1. Patients' phenotype and genotype, duration of miglustat treatment and follow-up, latency between onset of neurological manifestations and start of treatment.

Patient code	Sex	Phenotype	Gene	Genotype		Age at enroll-ment	Duration of previous miglustat therapy at enrollment (months)	Duration of miglustat exposure (months)	Age at onset of neurological symptoms (years)	Age at onset of visceral symptoms (years)	Age at onset of psychiatric symptoms (years)	Age at diagnosis (years)	Age at start of treatment (years)	Latency between onset of neurological symptoms and start of treatment (years)
				**allele 1**	**allele 2**									
A01	F	A	NPC1	c.1907C>T (p.S636F)	ND	18.83	0	60	16.00		16.00	18.00	19.00	3.00
A02	F	A	NPC1	c.2932C>T (p.R978C)	c.882-28A>G	29.90	23	96	19.00			25.00	28.00	9.00
A03	M	A	NPC2	c.26T>C (p.L9P)	c.26T>C (p.L9P)	43.83	0	60	33.00	39.00		43.83	43.83	11.00
A10	F	A	NPC1	c.3019C>G (p.P1007A)	c.3493G>A (p.V1165M)	28.83	0	48	19.00		24.00	28.00	28.83	9.83
A18	F	A	NPC1	C.1415T>A (p.L472H)	C.3230G>A (p.R1077Q)	40.10	0	48	26.00			29.66	40.10	5.80
A19	M	A	NPC1	c.2932C>T (p.R978C)	c.882-28A>G	33.75	23	96	18.00			26.00	31.80	13.80
J04	F	J	NPC1	c.3182 T >C (p.I1061T)	c.3182 T >C (p.I1061T)	15.66	31	96	6.00			7.70	13.00	7.00
J06	F	J	NPC1	c.2762A>C (p.Q921P)	c.2903A>G (p.N968S)	18.66	0	60	8.00			12.50	18.83	10.83
J07	M	J	NPC1	c.3182 T >C (p.I1061T)	c.3182 T >C (p.I1061T)	19.60	31	96	11.00	0.50		11.50	19.60	8.60
J11	M	J	NPC1	c.3493G>A (p.V1165M)	c.58-3T>G (spl?)	17.83	7	72	6.00			9.00	17.25	11.25
J12	F	J	NPC1	c.2762A>C (p.Q921P)	c.2903A>G (p.N968S)	13.33	0	54	10.00			12.70	13.25	3.25
J13	F	J	NPC1	c.2762A>C (p.Q921P)	c.2903A>G (p.N968S)	16.66	0	54	16.00			16.00	16.60	0.60
J15	F	J	NPC1	c.2800C>T (p.R934X)	ND	10.30	9	84	9.00	9.00		9.00	9.41	0.41
J16	F	J	NPC1	c.2800C>T (p.R934X)	ND	13.50	18	84	8.00	11.00		11.00	12.00	4.00
J20	M	J	NPC1	c.1421C>T (p.P474L)	c.1421C>T (p.P474L)	18.83	0	84	6.00	6.00		13.00	17.83	11.83
LI05	F	LI	NPC1	c.3493G>A(p.V1165M)	ND	9.75	0	48	4.00			9.00	9.25	5.25
LI08	F	LI	NPC1	c.3019C>G (p.P1007A)	c.3614C>A (p.T1205K)	7.75	0	60	2.50	0.01		7.50	7.75	5.25
LI09	F	LI	NPC1	c.3182 T >C (I1061T)	c.2762A>C (p.Q921P)	11.00	18	84	3.00	0.20		0.25	9.58	6.58
LI17	F	LI	NPC1	c.2829C>G (p.I943M)	ND	11.40	6	76	4.00	0.08		10.60	10.91	6.91
LI21	M	LI	NPC1	c.2339T>G (p.V780G)	c.2762A>C (p.Q921P)	3.10	0	66	2.00	0.01		2.80	3.00	1.00
LI25	F	LI	NPC1	c.2662C>T (p.P888S)	c.2761C>T (p.Q921X)	15.66	0	66	4.00	0.01		10.00	16.70	12.70
EI22	M	EI	NPC2	c.58G>T (E20X)	c.58G>T (E20X)	2.66	21	96	0.25	0.01		0.66	0.91	0.66
EI24	M	EI	NPC1	c.852delT (p.F284LfsX25)	c.852delT (p.F284LfsX25)	2.75	0	48	2.00	0.01		2.60	2.75	0.75
V14	F	NA	NPC1	c.3056A>G (p.Y1019C)	c.3056A>G (p.Y1019C)	2.33	22	84	absent	0.01		0.6	0.6	0.00
V23	M	NA	NPC1	c.3019C>G (p.P1007A)	c.3614C>A (p.T1205K)	1.58	0	60	absent	0.08		1.30	1.60	0.00

A= adult; J = Juvenile; LI = Late Infantile; EI = early infantile; V = visceral; ND = not detected
