# Supplementary material for: Long term follow-up to evaluate the efficacy of miglustat treatment in Italian patients with Niemann-Pick disease type C
Source: Orphanet J Rare Dis. 2015 Feb 27;10:22. doi: 10.1186/s13023-015-0240-y (PMC4359492; doi:10.1186/s13023-015-0240-y)
Supplement: Additional file 2: — Severity rating scale of neurological manifestations and dysphagia. [file 13023_2015_240_MOESM2_ESM.pdf]

Additional file 2. Severity rating scale of neurological manifestations and dysphagia

GAIT ABNORMALITIES	DEFINITION	Original score	Modified score
Absence of abnormalities	Normal ambulation	0	0
Mild abnormalities	Autonomous ataxic gait	1	0.33
Moderate abnormalities	Assisted ambulation	2	0.67
Severe/ invalidating abnormalities	Wheelchair bound	3	1

DYSMETRIA	DEFINITION	Original score	Modified score
Absence of abnormalities	Normal	0	0
Mild abnormalities	Mild difficulties in reaching the nose or an object	1	0.33
Moderate abnormalities	Moderate difficulties in reaching the nose or an object	2	0.67
Severe/ invalidating abnormalities	Severe dysmetria, preventing the patient from reaching nose or an object	3	1

DYSTONIA	DEFINITION	Original score	Modified score
Absence of abnormalities	Normal	0	0
Mild abnormalities	Slight dystonia, only with actions, not disabling/interfering with activities	1	0.33
Moderate abnormalities	Dystonia on action of distant part of body or intermittently at rest, interfering with normal activities and causing moderate impairment of function	2	0.67
Severe / invalidating abnormalities	Severe dystonic movements present at rest and/or causing complete impairment of function	3	1

DYSARTHRIA	DEFINITION	Original score	Modified score
Absence of abnormalities	Normal language	0	0
Mild abnormalities	Understandable language	1	0.33
Moderate abnormalities	Poor comprehensive language	2	0.67
Severe/ invalidating abnormalities	Non verbal communication /anarthria	3	1

DEVELOPMENTAL DELAY/ COGNITIVE IMPAIRMENT	DEFINITION	Original score	Modified score
Absence of cognitive impairment/psychomotor delay	Qualitative assessment reveals acquisition of normal milestones of psychomotor development or the patient is self-sufficient without any support.<br>Alternatively, IQ higher than 70 with two subcategories as follows:<br>- Absent intellectual disability: IQ higher than 84<br>- Borderline intellectual disability: IQ between 70 and 84.	0	0
Mild cognitive impairment/psychomotor delay	Qualitative assessment reveals mild delay in acquisition of psychomotor development milestones or the patient is fairly self-sufficient with minimal/intermittent support and has communication skills.<br>Alternatively, IQ between 50 and 69	1	0.33
Moderate cognitive impairment/psychomotor delay	Qualitative assessment reveals moderate delay in acquisition of psychomotor development milestones or the patient is able of self-care tasks with limited/moderate support and has some communication skills<br>Alternatively, IQ between 35 and 49	2	0.67
Severe cognitive impairment/psychomotor delay	Qualitative assessment reveals severe delay in acquisition of psychomotor development milestones or the patient is able / completely unable of very basic self-care skills with extensive or pervasive support and/or is able / completely unable of very basic communication skills<br>Alternatively, IQ below 35 with two subcategories as follows:<br>- Severe intellectual disability: IQ between 20 and 34<br>- Profound intellectual disability: IQ below 20	3	1

DYSPHAGIA	DEFINITION	Original score	Modified score
Normal swallowing	Absence of difficulty to swallow	0	0
Mild difficulty to swallow	Long feeding times, difficulty in chewing	1	0.25
Moderate difficulty	Arching or stiffening of the neck during feeding, gurgly, hoarse, or breathy voice quality	2	0.50
Severe difficulty	Excessive drooling or food/liquid coming out of the mouth or nose, coughing or gagging during meals, difficulty breast feeding	3	0.75
Unable to swallow	Refusing liquid or food of different texture, Nasogastric tube or gastric button feeding (PEG)	4	1
