# Supplementary figures and images for: Long term follow-up to evaluate the efficacy of miglustat treatment in Italian patients with Niemann-Pick disease type C
Source: Orphanet J Rare Dis. 2015 Feb 27;10:22. doi: 10.1186/s13023-015-0240-y (PMC4359492; doi:10.1186/s13023-015-0240-y)

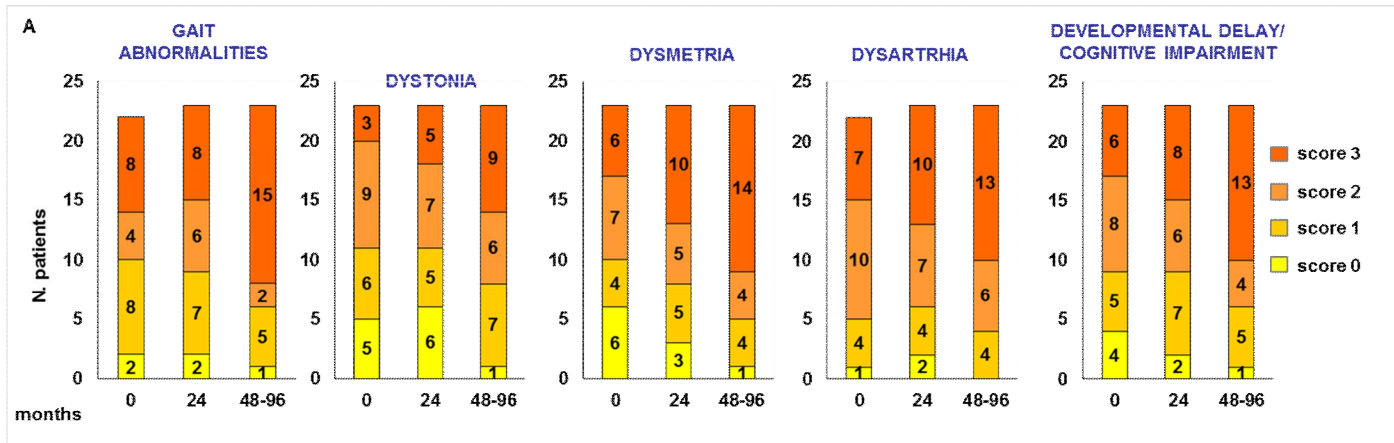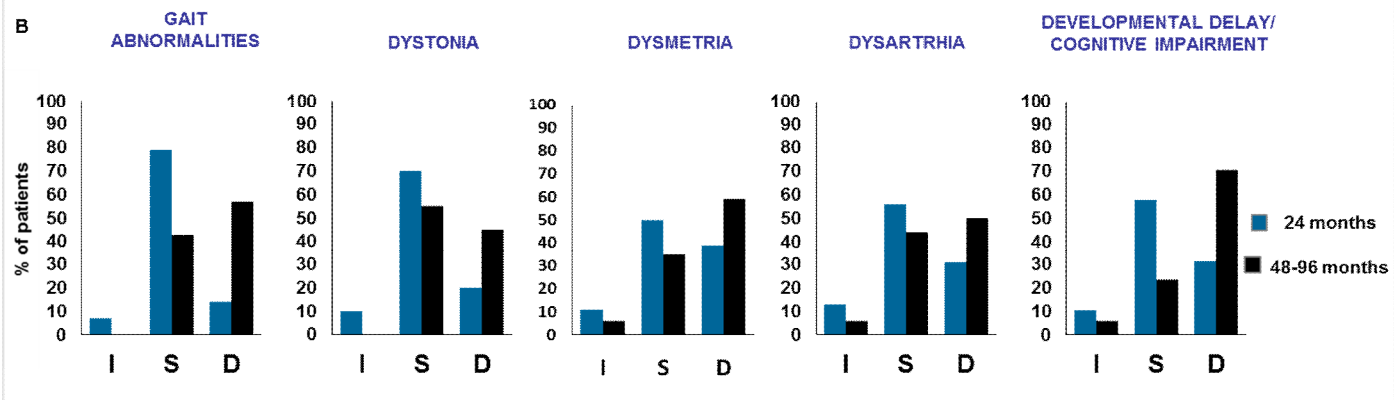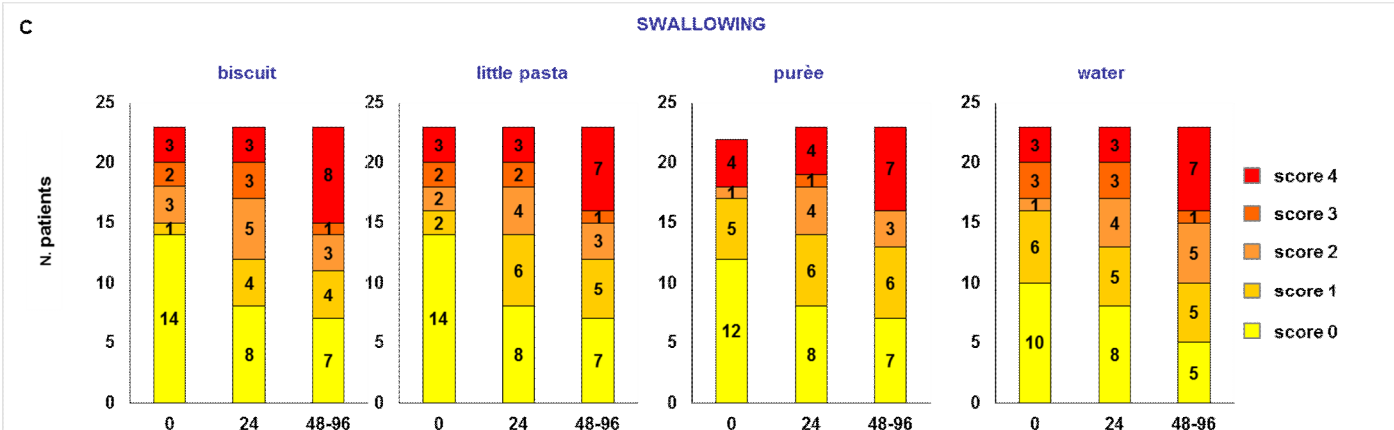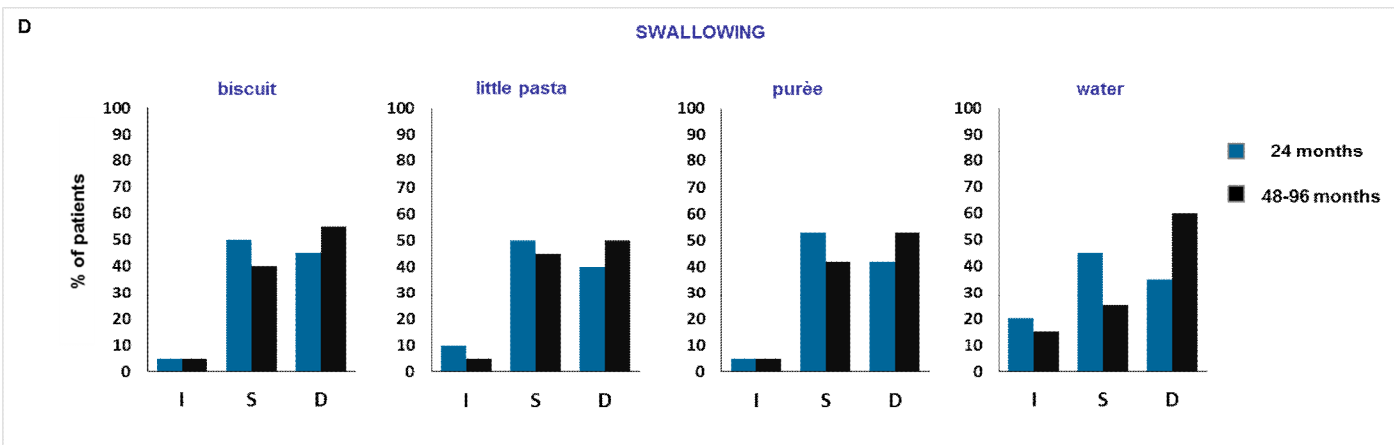

Supplement: Additional file 3: — Distribution of severity score of five neurological parameters at baseline and after 24 and 48–96 months of treatment. A: The severity score distribution for gait abnormalities, dystonia, dysmetria, dysarthria and developmental delay/cognitive impairment showed heterogeneous clinical conditions in enrolled patients at baseline B: Modification of severity score of five neurological parameters, compared to baseline, after 24 and 48–96 months of treatment. After 24 months of treatment most patients showed improvement or stabilization of the severity score for gait abnormalities, dystonia, dysmetria, dysarthria and developmental delay/cognitive impairment. C: Distribution of severity score for swallowing of four different substances at baseline and after 24 and 48–96 months of treatment. The severity score distribution for swallowing ability showed heterogeneous clinical conditions in enrolled patients at baseline. D: Modification of severity score for swallowing of four different substances, compared to baseline, after 24 and 48–96 months of treatment. After 24 months of treatment most patients showed improvement or stabilization of the ability to swallow four substances with different consistencies. Abbreviations: I = improvement; S = stabilization; D = deterioration. [file 13023_2015_240_MOESM3_ESM.pdf]

LAG  $\leq 3,5$  years

a

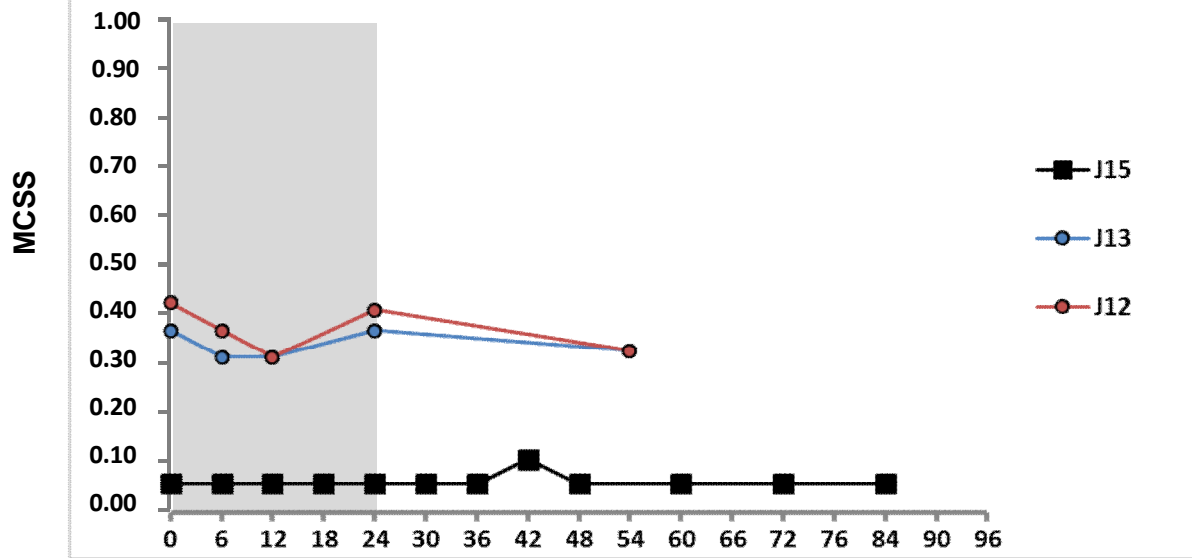

LAG  $\geq 3,5$  years

b

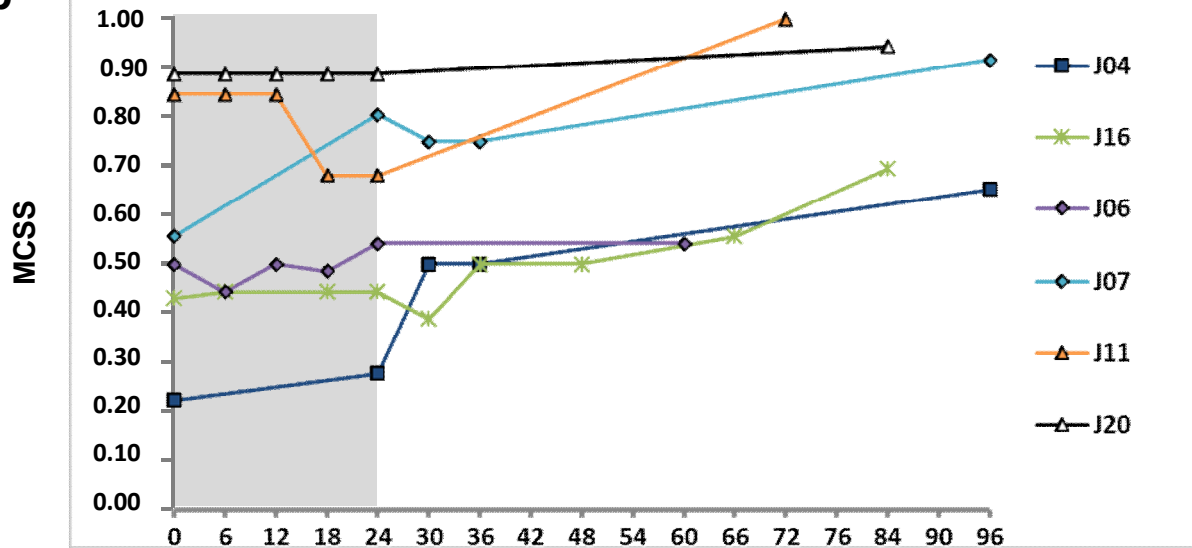

Supplement: Additional file 4: — Evolution over time of the mean composite severity score (MCSS) during miglustat treatment in juvenile patients, based on the latency between the onset of neurological manifestations and start of therapy (LAG). Patients with a) LAG ≤ 3.5 years (n = 3); b) LAG >3.5 years (n = 6). [file 13023_2015_240_MOESM4_ESM.pdf]

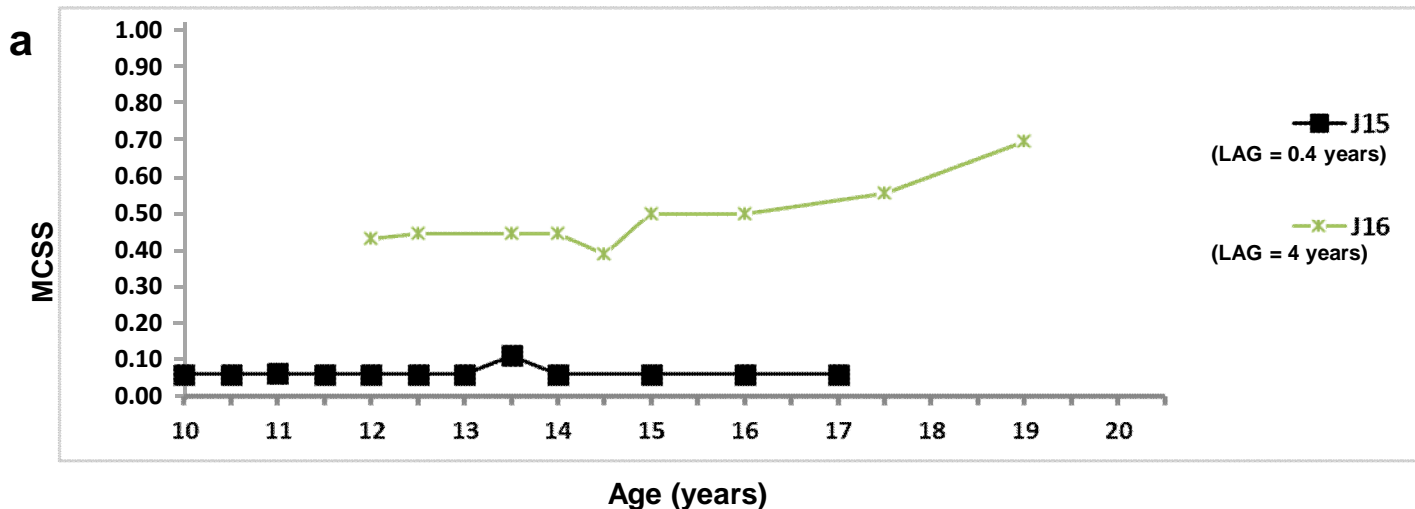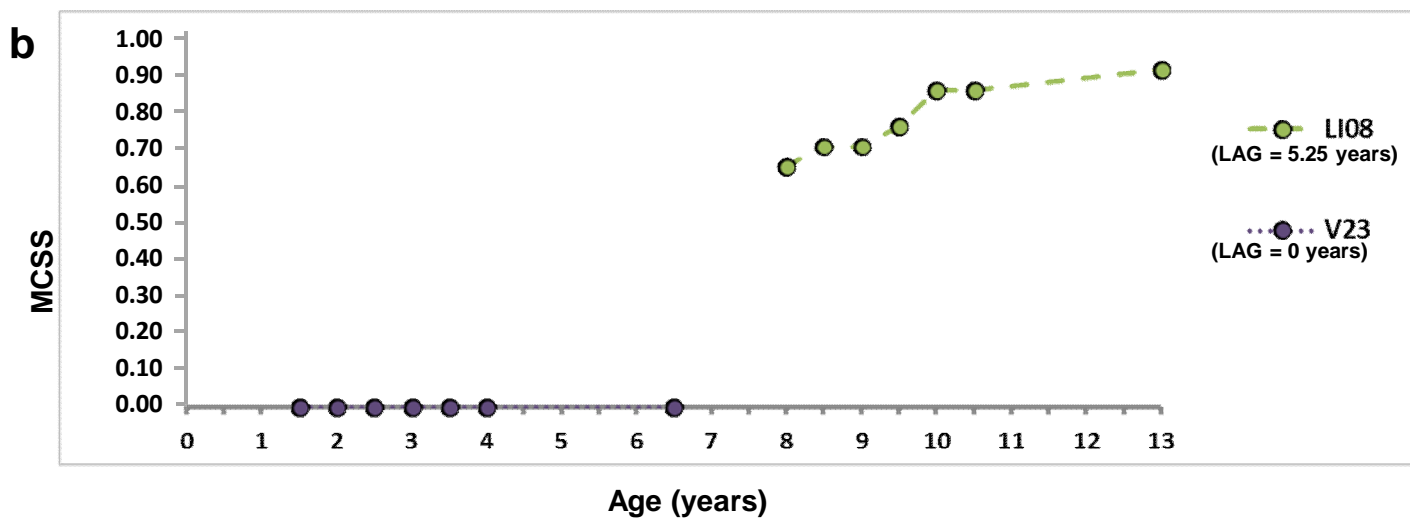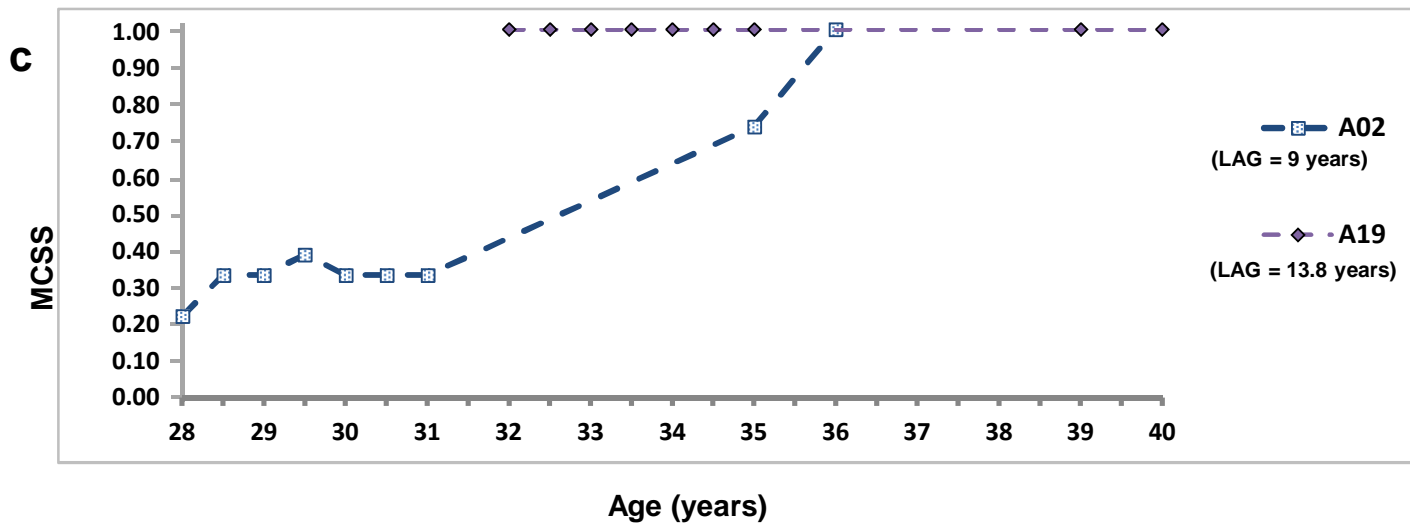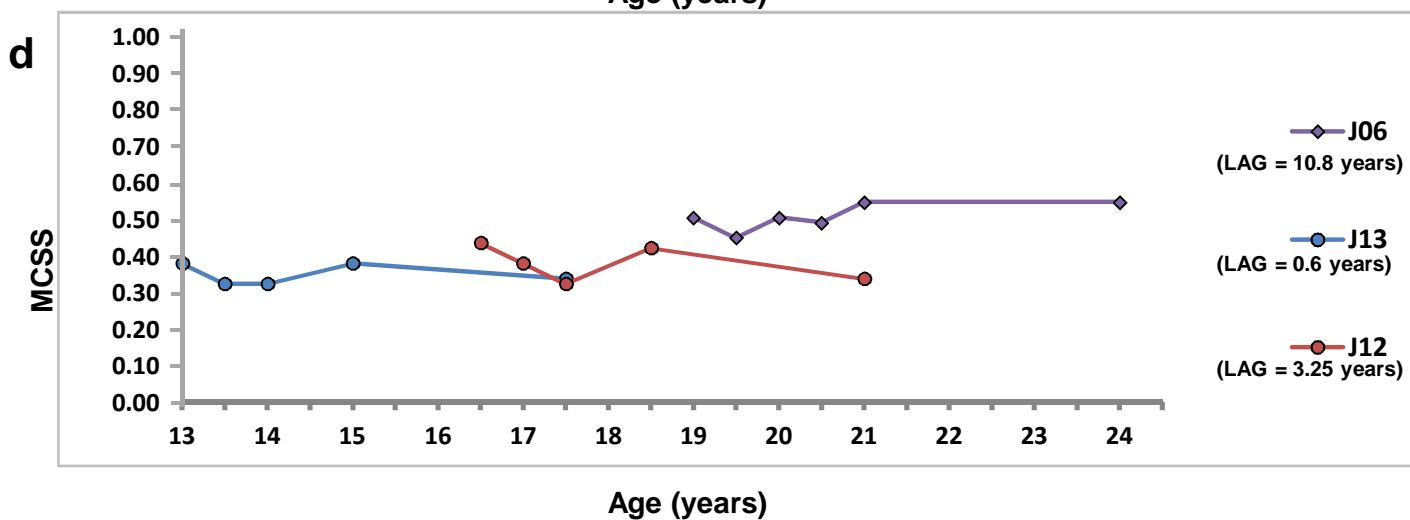

Supplement: Additional file 5: — Evolution over time of the mean composite severity scores (MCSS) in groups of siblings. In sections a) and b) siblings were treated with different latency between the onset of neurological manifestations and start of therapy (LAG); in section c) siblings were both treated with LAG > 7 years. In section d) both patients J12 and J13 were treated early (LAG < 3,5 years), while patient J06 had a longer latency. [file 13023_2015_240_MOESM5_ESM.pdf]
